# Dissociating polysensitization and multimorbidity in children and adults from a Polish general population cohort

**DOI:** 10.1186/s13601-019-0246-y

**Published:** 2019-02-11

**Authors:** Filip Raciborski, Jean Bousqet, Andrzej Namysłowski, Edyta Krzych-Fałta, Aneta Tomaszewska, Barbara Piekarska, Piotr Samel-Kowalik, Artur Z. Białoszewski, Artur Walkiewicz, Agnieszka Lipiec, Oksana Wojas, Krzysztof Samoliński, Anna Szylling, Wojciech Zieliński, Adam Sybilski, Aleksandra Grąbczewska, Bolesław Samoliński

**Affiliations:** 1 Department of Prevention of Environmental Hazards and Allergology, Medical University of Warsaw, Warsaw, Poland; 2 MACVIA-France and Fondation FMC VI-LR, Montpellier, France; 3 Ageing and Chronic Diseases, Epidemiological and Public Health Approaches, U1168, INSERM, VIMA, Paris, France; 4 UMR-S 1168, Université Versailles St-Quentin-en-Yvelines (UVSQ), Paris, France; 5 Euforea, Brussels, Belgium; 6 Charité, Berlin, Germany; 7 Allergy and Clinical Immunology Department, Central Hospital Medical University of Warsaw, Warsaw, Poland; 8 Public Health Department, Medical University of Warsaw, Warsaw, Poland; 9 Department of Econometrics and Statistics, Warsaw University of Life Sciences, Warsaw, Poland

**Keywords:** Muitimorbidity, Polysensitization, IgE, Skin tests, Allergy, Asthma, Rhinitis, Urticaria, Atopic dermatitis

## Abstract

**Background:**

Links between multimorbidity of allergic diseases and allergen sensitization are still under debate, especially in adults. This study aimed to establish a relationship between polysensitization and allergic multimorbidity in children and adults and the allergens involved in multimorbidity.

**Material and method:**

A cross-sectional multicentre study enrolled children aged 6–7 and 13–14 years and adults aged 20–44 years from a Polish national cohort. The diagnosis of allergic diseases was made by a physician. Skin prick tests to 13 allergens and serum IgE levels to 4 allergens were tested.

**Results:**

Among the 3856 participants, single disease (asthma, allergic rhinitis or atopic dermatitis) was diagnosed in 27.7% subjects and allergic multimorbidity in 9.3%. Allergic multimorbidity occurred more commonly in children than in adults (p < 0.01). Asthma or atopic dermatitis alone were not associated with polysensitization. Rhinitis and multimorbidity were associated with polysensitization. Allergic multimorbidity occurred in 2.2% of participants with negative skin prick tests, 9.8% of those with one positive prick test (SPT ≥ 3 mm) and 20.6% of polysensitized ones (p < 0.001). There was an increasing risk of multimorbidity depending on the number of positive prick tests for both SPT ≥ 3 mm (OR 9.6–16.5) and SPT ≥ 6 mm (OR 5.9–13.7). A statistically significant relationship was found between allergic multimorbidity and sensitization to cat and mite allergens.

**Conclusions:**

Multimorbidity is associated with polysensitization especially in children compared with adults in Polish population cohort. New insights into single disease patterns were found: bronchial asthma is the strongest risk factor for the development of multimorbidity in comparison with allergic rhinitis and atopic dermatitis.

**Electronic supplementary material:**

The online version of this article (10.1186/s13601-019-0246-y) contains supplementary material, which is available to authorized users.

## Introduction

Asthma (A), rhinitis (Rh) and atopic dermatitis (AD) are common chronic diseases. One of the most challenging characteristics is their complexity, with multiple genetic [1–5] and environmental factors interlinked through IgE- and non-IgE-associated mechanisms [6]. These diseases generally begin very early in life and may persist across the life cycle [7]. They co-occur in the same subjects (multimorbidity) more often than expected by chance [5]. Multimorbidity is associated with IgE and non-IgE mechanisms. Moreover, the co-occurrence of these conditions appears to differ for various types of allergy [8].

There are several gaps in our knowledge. (i) Allergic multimorbidity has been studied in children and, more rarely, in adults [9]. However, no epidemiologic study has considered multimorbidity from childhood to adulthood. (ii) The type of allergic sensitization linked with multimorbidity is not yet fully understood. Asthma usually occurs less commonly in seasonal or intermittent allergic rhinitis (AR) in comparison to persistent allergy [1, 2, 10–12]. The relationship between atopic dermatitis and AR or A needs closer investigation in adults [12]. The role of urticaria, food or insect allergy is unknown in allergic multimorbidity, [11–15].

The phenotypic characterization of allergic diseases and the interactions between AR and A with sensitization suggests the importance of better characterization of subjects with allergic diseases and sensitisation to specific allergens for both clinical practice and mechanistic studies [16]. These studies are likely to impact the political agenda outlined by the Conclusion of the European Council on respiratory diseases among children [17–19].

## Objectives

The aim of this study was to establish (i) a relationship between polysensitization and allergic multimorbidity in children and adults, (ii) the impact of other allergic and related diseases on multimorbidity and (iii) the allergens associated with single disease or multimorbidity.

## Methods

The paper has been written according to the Strengthening the Reporting of Observational Studies in Epidemiology (STROBE) checklist (www.strobe-statement.org) [20].

### Study design

The project Epidemiology of Allergic Diseases in Poland (ECAP) is an allergic epidemiological study conducted in Poland. It consists of two phases: an initial fieldwork phase (research grant from Ministry of Health and Ministry of Science and Higher Education (Poland) 03788/C.P05-6/2005) and a second laboratory-based study (research grant from the National Science Centre (Poland), 2011/01/B/NZ7/05289).

### Setting

The ECAP is a cross-sectional multicentre study. The project includes three age categories: (i) children aged 6–7 and (ii) 13–14 years and (iii) adults aged 20–44 years living in the eight largest Polish urban centres (Warsaw, Lublin, Białystok, Gdańsk, Poznań, Wrocław, Katowice, Krakow) and one rural area (Zamość and Krasnystaws districts) (Additional File 1: Fig. 1).

The study comprised two main parts: (i) a questionnaire-based study (Computer-Assisted Personal Interview-CAPI); (ii) a complementary clinical assessment (spirometry with bronchodilator challenge, skin-prick tests (SPT), peak nasal inspiratory flow, and blood sampling for genetic and immune tests) [21]. Every third respondent participating in the questionnaire was recruited in a randomised procedure to participate in the complementary clinical assessment.

### Participants

The stratified cluster sampling method was used to draw the research sample in each of the 9 centres. The sample was drawn from a personal identity number (PESEL) database (maintained by the Minister of Interior and Administration) which covers the entire population of Polish residents. Detailed information about the sampling and research methods is available on the ECAP website (http://ecap.pl/pdf/ECAP_metoda.pdf) [22].

### Data sources—measurement

#### Clinical examination

During phase 2 of the study, each respondent was examined by a specialist in allergology who had participated in a training to harmonize clinical diagnoses. In adults, the diagnosis of A and Rh followed the pan-European study European Community Respiratory Health Survey II (ECRHS II) [23]. In children, the protocol and methodology of the International Study of Asthma and Allergy in Childhood (ISAAC) were used [24] without any modification. For AD, the criteria of Hanifin and Rajki were used [25].

#### Skin-prick test

Participants of phase 2 had a SPT [23] with the following 13 allergens: birch, grass pollen, mugwort, *Dermatophagoides pteronyssinus* (Der p), *Dermatophagoides Farinae* (Der f), dog, cat, hazel, alder, rye grass, plantain, *Cladosporium, Alternaria* negative control, histamine (Allergopharma, Reinbek, Germany). Single drops of allergen extracts (Allregopharma-Nexter) were administered at 2–5 cm-intervals using standard Morrow-Brown needles (0.9 mm long blade). The puncture was performed at a depth of approximately 0.4 mm and 0.01–0.5 μl of allergen extracts were administered into a skin (expressed in biological unit BU/ml). The interpretation of skin prick test results (based on the guidelines of the European Academy of Allergy and Clinical Immunology [26]) was made after approximately 15–20 min in relation to positive (the histamine dihydrochloride at a concentration of 1.0 mg/ml) and negative control (the saline). If the diameter of oedema was > 3 mm, it indicated that a specific IgE for a given allergen was present in the skin. Each patient was skin tested on the volar surface of the forearm using 1-mm prick lancets. The skin reaction was recorded after 15 min by evaluating the skin response in comparison with the wheal produced by the positive and negative controls. A wheal diameter of at least 3 mm is considered a positive reaction according to the EAACI (European Academy of Allergy and Clinical Immunology) recommendation [26].

Due to cross reactions between allergens, the allergens were combined into 8 groups: (i) dog, (ii) cat, (iii) *Cladosporium herbarum*, (iv) *Alternaria tenuis*, (v) Hazel, Alder and Birch, (vi), Der p and Der f (vii) Grass pollen and Rye grass, (viii) Mugwort and Plantain, although these weed pollens are not cross-reactive.

Positive SPT readings were recorded as weakly positive (SPT ≥ 3 mm) or strongly positive (SPT ≥ 6 mm).

#### Specific IgE

Serum specific IgE (sIgE) to d1—*D pteronyssinus*, g6—*Phleum pratense*, m6—*Alternaria alternata*, and e1—cat was assayed by the CAP method (Thermofisher, Uppsala, Sweden). The following two thresholds were set for slgE values: ≥ 0.35 IU/ml (class ≥ 1) and ≥ 0.7 IU/ml (class ≥ 2).

### Variables

The following definitions were adopted: **allergic multimorbidity** is the co-occurrence of at least two of the following three conditions: A, AR and AD. Other allergic diseases (e.g. food and venom allergy, and urticaria) were analysed but they were not included in the multimorbidity definition. **Polysensitization** is sensitization to at least two allergens or groups of allergens (sensitization to two allergens within the same group is not considered as polysensitization).

### Biases

One of the problems of studies with two phases is the proportion of participants willing to have a complementary study. In the group of children (6–7 years) and adolescents (13–14 years), the attendance rate was higher than in the group of adults. Subjects with symptoms of allergic diseases were more willing to take part in the clinical assessment.

### Statistical methods

The following statistical methods were used: descriptive analysis (e.g. frequencies, mean, median), bivariate analysis (crosstabs), multivariate analysis (logistic regression). The results were presented as odds ratio (including forest plot), confidence interval and p-value (Pearson’s Chi squared). Multivariable regression model was used to reduce the potential impact of confounding factors.

Statistical analysis was restricted to subjects with complete data on the variables involved in the analysis. No data imputation method was involved.

All analyses were performed using IBM SPSS version 23 and 24. A significance level of 0.05 was assumed.

## Results

### Participants

**Fig. 1:**
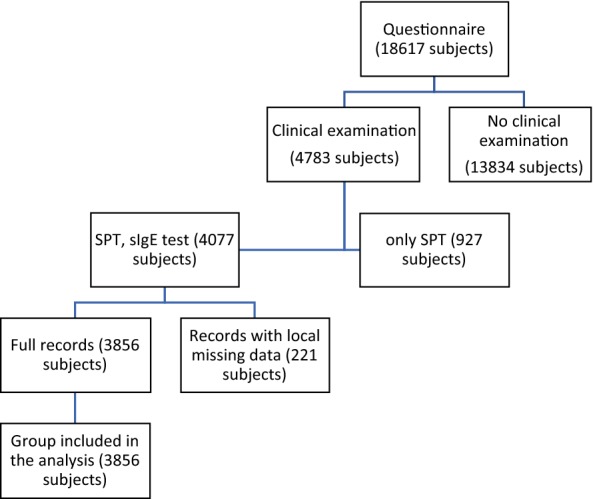
STROBE flow chart

The study group comprised 3856 subjects with complete clinical examination (including SPT and sIgE tests results) and questionnaire data (Fig. 1).

Characteristics of the study group are presented in Table 1 and Additional file 1: Table 1.

**Table 1:** Characteristics of the study group (% in columns)

	Total	
	N	%
Total	3856	100
Age		
6–7 years	967	25.1
13–14 years	1084	28.1
20–24 years	457	11.9
25–29 years	353	9.2
30–34 years	350	9.1
35–39 years	320	8.3
40–44 years	325	8.4
Gender		
Female	2102	54.5
Male	1754	45.5
Allergic diseases		
Asthma	415	10.8
Asthma alone (without AR, AD)	138	3.6
Allergic rhinitis	1130	29.3
Allergic rhinitis alone (without A, AD)	790	20.5
Atopic dermatitis	278	7.2
Atopic dermatitis alone (without A, AR)	142	3.7
Food allergy	344	8.9
Drug allergy	165	4.3
Insect venom allergy	94	2.4
Urticaria	247	6.4
Positive SPT (≥ 3 mm)		
Control	31	0.8
Histamine	3617	93.8
Dog	346	9.0
Cat	512	13.3
Cladosporium	212	5.5
Alternaria	331	8.6
Hazel & Alder & Birch	753	19.5
*D. pteronyssinus* & *D. farinae*	1039	26.9
Grass pollen & Rye grass	904	23.4
Mugwort, Plantain	738	19.1
Positive sIgE (≥ 1 class)		
d1—*D. pteronyssinus*	563	14.7
g6—*Phleum pratense*	509	13.3
m6—*Alternaria alternata*	149	3.9
e1—cat	235	6.1
SPTs^a^		
All 8 SPTs negative	2158	56
Only 1 of 8 SPTs ≥ 3 mm	583	15.1
Polysensitisation (≥ 3 mm)	1115	28.9
All 8 SPTs < 6 mm	2960	76.8
Only 1 of 8 SPTs ≥ 6 mm	506	13.1
Polysensitisation (≥ 6 mm)	390	10.1
sIgE^b^		
All sIgE in class 0	2885	74.8
1 of 4 sIgE ≥ class 1	621	16.1
Polysensitisation (≥ class 1)	350	9.1
All sIgE class in 0 or 1	3032	78.6
1 of 4 sIgE ≥ class 2	571	14.8
Polysensitisation (≥ class 2)	253	6.6

*AR* allergic rhinitis, *AD* atopic dermatitis

^a^8 groups of allergens

^b^4 allergens

### Descriptive data

Asthma was diagnosed in 10.8% of the participants, AR in 29.3% and AD in 7.2% (Table 2). Asthma without AR or AD was found in 3.6% of the subjects. AR without A or AD was diagnosed in 20.5% of the subjects, and AD without A or AR in 3.7% (Additional file 1: Table 2). A single disease (A, AR or AD) was observed in 27.7% of the subjects and allergic multimorbidity in 9.3% (8.4% had dual morbidity, 0.9% had triple morbidity.

Allergic multimorbidity occurred in 10.7% of the 6–7-year-old children, in 10.9% of the 13–14-year-old children and in 7.6% of the adults (p < 0.01). The prevalence of allergic multimorbidity was higher among men (10.5%) than women (8.3%) (p < 0.05) (Additional file 1: Fig. 2).

Positive SPTs (≥ 3 mm) to at least 1 group of allergens were seen in 44% of the study group. A strongly positive reaction (SPT ≥ 6 mm) to at least 1 group of allergens was present in 23.2%. Among subjects without any positive SPT, allergic multimorbidity occurred in 2.3%, compared to 9.4% of those with one positive SPT (≥ 3 mm) and 22.9% of polysensitized (SPT ≥ 3 mm) ones (p < 0.001) (Additional file 1: Fig. 2).

Positive SPTs (≥ 3 mm) was most often observed for dust mites (*Der p and Der f:* 26.9%) and grass pollen (23.4%). A positive (class ≥ 1) slgE to *Der p* was observed in 14.7% subjects, to cat in 6.1%, to *Phleum pratense* in 13.3% and to *Alternaria tenuis* in 3.9%.

### Main results: relationship between polysensitization and multimorbidity

Of the conditions included in the definition of allergic multimorbidity, asthma had the greatest impact on the risk of multimorbidity (OR = 82.2, 95% CI 60.9–110.9) compared to AR (OR = 61.3, 95% CI 38.4–98.0) and AD (OR = 14.4 95% CI 10.9–18.9).

**Fig. 2:**
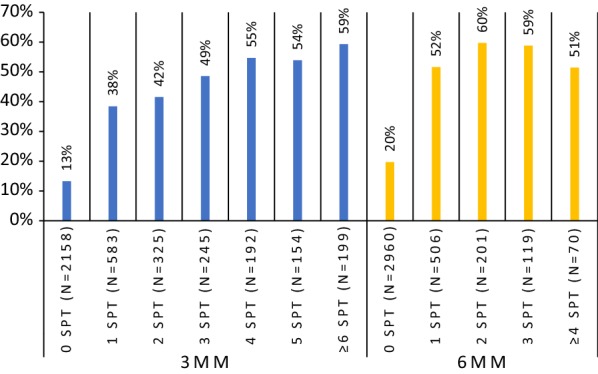
Percentage of subjects with allergic multimorbidity by the number of positive SPTs (8 groups of allergens). X axis—number of positive allergen skin prick tests. Y axis—% of subjects with allergic multimorbidity

**Fig. 3:**
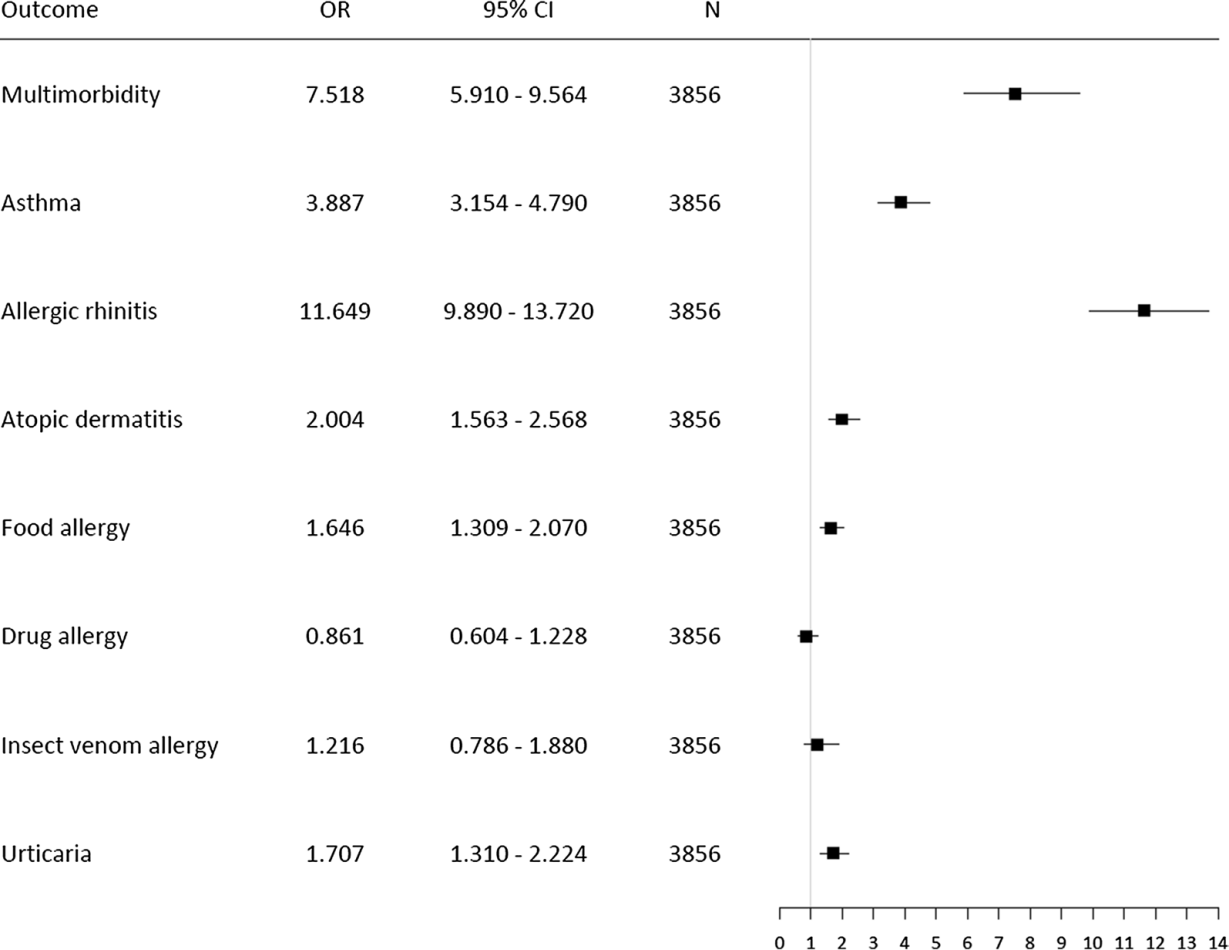
The influence of diseases on risk of allergic polysensitization assessed by skin prick tests

The occurrence of multimorbidity increased with the number of positive SPTs (Fig. 2). Allergic multimorbidity was strongly associated with polysensitization as assessed by skin tests (OR = 7.5, 95% CI 5.9–9.6). The highest association occurred between polysensitization and AR. A moderate relationship was found with A, AD, food allergy and urticaria (OR 2.0–3.9) (Fig. 3 and Additional file 1: Table 3).

**Fig. 4:**
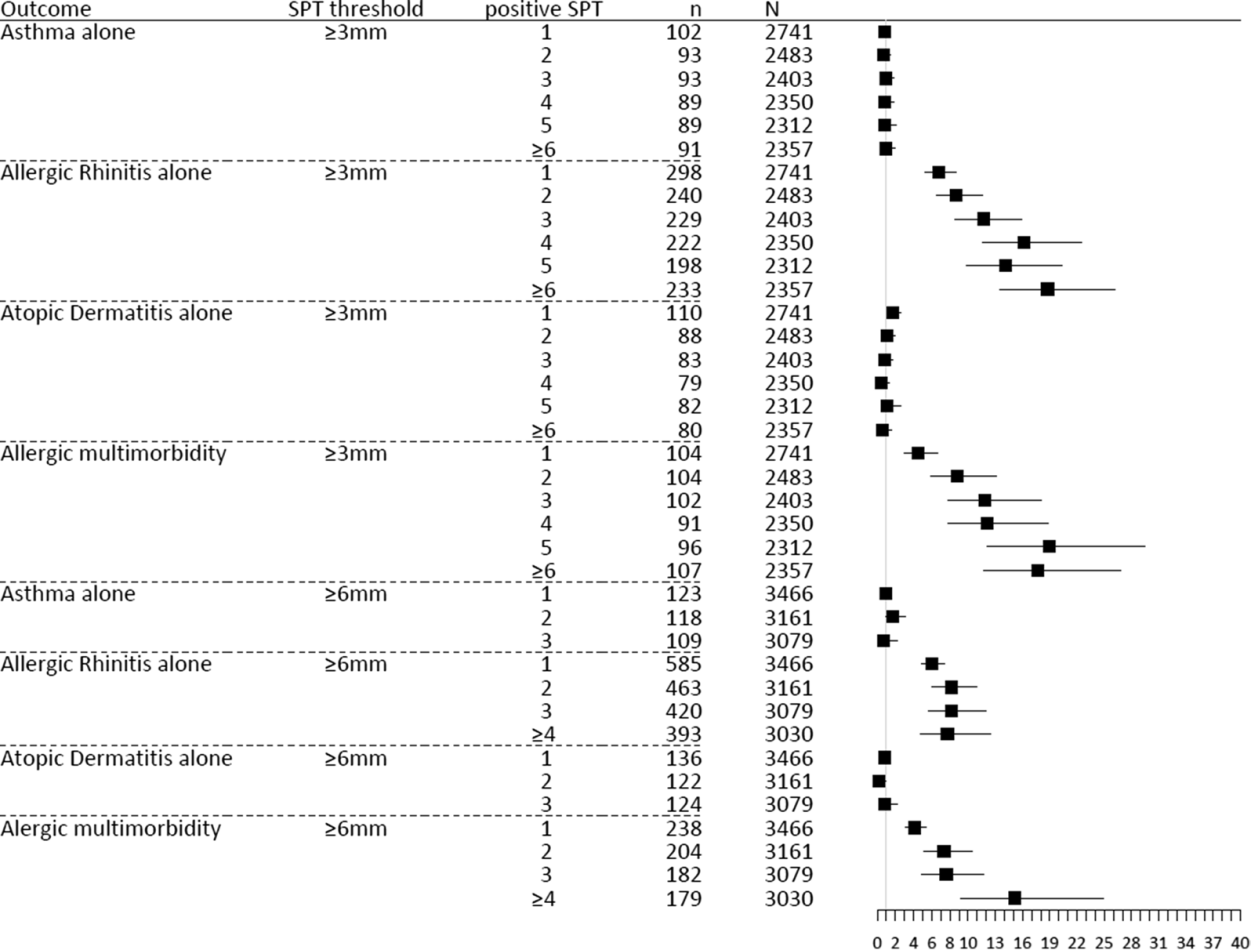
Association between allergen sensitization for ≥ 3 mm and ≥ 6 mm SPT threshold and allergic diseases. SPT—allergen skin prick test. n—number of subjects with a confirmed diagnosis. N—total number of subjects in the analysis (with 0 positive SPT or x SPT, where x is number of positive SPT)

Figure 4 and Additional file 1: Table 4 show that there was no statistically significant relationship (based on OR analysis) between A (as a single disease) and the number of positive SPTs (for both the 3 mm and 6 mm threshold). Results for AD were similar with two exceptions (for 1 SPT (3 mm) and 2 SPTs (6 mm)). The risk of AR (as a single disease) increased with each additional positive SPT (OR 6.7–18.8 for SPT ≥ 3 mm and 6.0–8.2 for ≥ 6 mm). There was a relationship between allergic multimorbidity and the number of positive SPTs. For both thresholds (≥ 3 mm and ≥ 6 mm) a higher number of positive SPTs was associated with a higher risk of multimorbidity (OR 4.5–18.9 for the 3 mm threshold and 4.1–15.1 for 6 mm). Differences between ORs for monosensitization (SPT = 1) and polysensitization (SPT ≥ 2) were statistically significant for the 3 and 6 mm thresholds (p < 0.05).

**Fig. 5:**
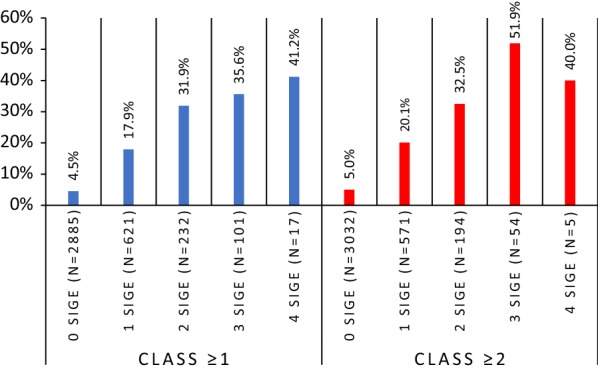
Percentage of people with allergic multimorbidity by the number of positive allergen-specific IgE assays (4 allergens: d1, e1, m6, g6). X axis—sIgE assay; Y axis—% of multimorbidity among those with positive sIgE for 0, 1, 2 and 3 allergens

A similar relationship between allergic multimorbidity and polysensitization was observed with sIgE levels. The prevalence of multimorbidity increased with the number of positive sIgE assays (Fig. 5 and Additional file 1: Fig. 2). This relationship was more pronounced in the analysis based on sIgE levels ≥ 0.70 IU/ml (class ≥ 2).

**Fig. 6:**
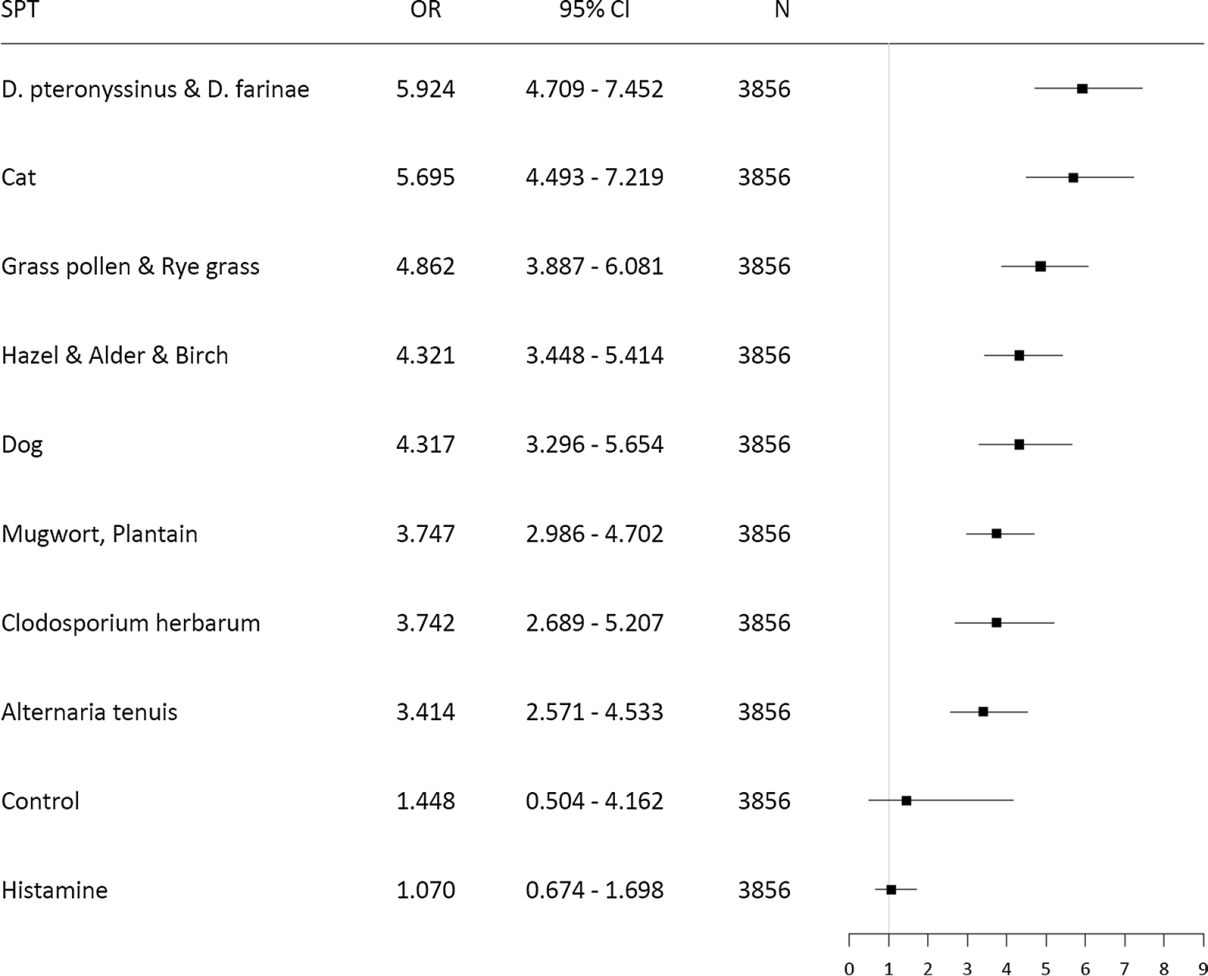
The importance of sensitization to particular allergens (positive SPT ≥ 3 mm) for the occurrence of multimorbidity (n = 3856)

The risk of multimorbidity also depended on the type of allergen, increasing from moulds (*Alternaria tenuis* OR 3.4, *Cladosporium herbarum* OR 3.7) to mites (OR 5.9) (Fig. 6).

### Multivariate analysis

The logistic regression model explaining multimorbidity was based on data on age categories, gender, co-occurrence of other types of allergies (except A, AR and AD) and SPT results. The -2 Log-likelihood value was 1933.896 (Cox & Snell R^2^ = 0.111, Nagelkerke’s R^2^ = 0.241). Among the variables analysed, there was a statistically significant relationship between multimorbidity and age categories (children and adolescents had a higher risk of multimorbidity than adults), food allergy and urticaria (higer risk), and positive SPTs for cat (high risk) and mites (high risk). The strongest impact on the risk of multimorbidity in the regression model was exerted by polysensitization for the threshold level of ≥ 3 mm. However, a clear impact was also seen in the case of monosensitisation (Table 2).

**Table 2:** The influence of different parameters on multimorbidity (logistic regression)

Name of variable	OR	95% CI	
		Lower	Upper
Age: 6–7 years	2.001	1.467	2.729
Age: 13–14 years	1.426	1.071	1.899
Sex: Male	1.033	0.812	1.314
Food allergy	2.397	1.726	3.328
Drug allergy	1.025	0.581	1.809
Insect venom allergy	1.464	0.774	2.770
Urtricaria	1.645	1.118	2.419
Dog (SPT)	1.149	0.832	1.585
Cat (SPT)	1.679	1.253	2.251
*Cladosporium herbarum* (SPT)	1.109	0.748	1.643
*Alternaria tenuis* (SPT)	1.010	0.726	1.406
Hazel & Alder & Birch (SPT)	1.058	0.785	1.426
*D. pteronyssinus* & *D. farinae* (SPT)	1.695	1.247	2.305
Grass pollen & Rye grass (SPT)	1.166	0.855	1.591
Mugwort, Plantain (SPT)	0.991	0.739	1.329
Monosensitisation (SPT)	3.512	2.289	5.388
Polysensitisation (SPT)	5.655	3.381	9.459
Constant	0.488		

Reference line: female, age 20–44, all SPTs negative

## Discussion

Allergy of chronic origin, especially in young children, manifests symptoms (of varying degrees of severity) from various organs/systems and is multifactorial in nature [27]. Multicentre cohort studies conducted in the population of young children showed strong coexistence between the response to more than 2 allergens and multiple morbidities, which concerned the co-occurrence of bronchial asthma, allergic rhinitis, eczema and allergic conjunctivitis [27, 28]. An association between multiple morbidities and multiple allergies was also observed in adults [15]. Our study involved both children and adults. In both these groups, an association between multiple morbidities and multiple allergies was found.

Multimorbidity is strongly associated with AR or A and, to a lesser extent, with AD. This paper offers novel findings and shows also association with food allergy and urticaria. Moreover, the paper brings other novel findings. Asthma or AD as single diseases are not associated with sensitization. Allergic rhinitis is associated with polysensitization but more so for SPT ≥ 3 mm. Multimorbidity is associated with polysensitization for SPT ≥ 3 and ≥ 6 mm. Cat is the allergen most strongly associated with multimorbidity.

### Strengths and limitations

The study used canonical epidemiologic methods with validated methods for the diagnosis of allergic diseases. Moreover, the diagnosis was made by trained physicians and incorrect diagnoses are unlikely.

Diagnosis of polysensitization in the study was based on a SPT (8 groups of allergens) or sIgE assay (4 antibodies).

Only three allergic diseases (A, AR, AD) were included in the multimorbidity analysis because (i) this is the classical approach to allergic multimorbidity and (ii) we wanted to assess the impact of other allergic diseases in multimorbidity. Consideration of other allergic diseases in multimorbidity (e.g. food allergy) might have led to different conclusions.

Among the subjects with diagnosed AR, 14.5% (15.9% for AR alone) had a negative SPT. These diagnoses were based on clinical history. These cases require further analysis.

Due to the multicentre character the study was representative for individual age groups in each of the analysed 9 centres, however, it was not representative at the country level.

### Interpretation of the findings

A, AR and AD were clearly associated with allergic multimorbidity. Also food allergy and urticaria increased the risk of multimorbidity. The role of venom allergy is more complex since the multivariable analysis showed no association, but according to a two-variable analysis it increased the risk. There was no association with drug allergy.

It is important to characterise multimorbidity across the life cycle. Although monosensitization and single allergic diseases are more common in infants, multimorbidity and polysensitization are found in preschool children [29–34]. The present study shows that multimorbidity is more common in children but also exists in adults, confirming the study of Siroux et al. [15].

Therefore it is essential to attempt to determine factors influencing the development of allergic multimorbidity. Ciprandi indicated that polysensitization co-occurs with multimorbidity [34]. Usually, polysensitization is more common than monosensitization, which was also demonstrated in this study (Table 1). Multimorbidity is also usually associated with a more severe course of AR and A. If it co-occurs with AD in adults, its clinical picture is usually characterised by severe, chronic dermatitis and respiratory tract inflammation [34, 35].

In the present study, the risk of multimorbidity was significantly associated with polysensitization. Multimorbidity was rare in case of negative SPTs, more frequent in subjects with one positive SPT reaction and very frequent in those with 2 or more positive SPT reactions to common inhalant allergens. Both SPT and slgE data were analysed, since according to epidemiologic studies, they show considerable overlap, but they do not have the same value for the interpretation of the allergic risk [36]. In cases of strongly positive reactions in SPT the significant association is present for at least 4 allergens.

Interestingly, A or AD alone were not associated with polysensitization (Fig. 4 and Additional file 1: Table 4). Polysensitization was associated with multimorbidity for SPT size of ≥ 3 or ≥ 6 mm. For AR, association with polysensitization was only shown for SPT ≥ 3 mm. These data suggest that A or AD alone, are different phenotypes in comparison to multimorbid A or AD. The data related to the size of the SPT reaction are compelling and may suggest differences depending on the size of the SPT. More studies are needed to understand this finding.

Moreover, attention should be paid to the significance of A in cases of multimorbidity. A was not the most common disease entity occurring in cases of multimorbidity, but if it was diagnosed in a patient, the probability of multimorbidity was significantly higher. The present results refer both to the group of patients diagnosed with allergic diseases and to the group of individuals without diagnosed allergies. This kind of analysis was conducted because both positive SPT reactions and the presence of slgE in serum might occur in people without allergy or, conversely, allergic symptoms might be present in patients with negative SPTs as suggested by topical allergy, e.g. Rh without antibodies in blood serum [37, 38]. This observation confirms the conclusion of Bousquet et al.: “Rhinitis is usually associated with mono- or polysensitization, whereas A is more often associated with polysensitization and multimorbidities” [30].

The present study was not focused on asymptomatic patients. However, it has to be noted that this phenomenon should be taken into account in further analysis and might be relevant in determining the significance of specific disease entities in the development of multimorbidity and polysensitization.

Not only polysensitization plays an important role in multimorbidity; the species of allergens are also important. Sensitization to cat or house dust mites (HDM) was more strongly associated with multimorbidity than to pollens or moulds. Although cat and HDM are perennial allergens, hazel and grasses are not. However, in the Polish climate these two allergens (together with birch and alder, which exhibit strong cross-reactivity with hazel) have a long pollen season from the end of January to April. These data suggest that long-acting allergens may play an important role in multimorbidity because of prolonged time of exposure.

Age plays some role in multimorbidity. The prevalence of multimorbidity was more common in children and adolescents than in subjects over 20 years of age.

### Generalisability

This study and analysis prove that multimorbidity of allergic diseases is a different phenotype compared to A alone and AD alone. Asthma plays a crucial role in multimorbidity but only with coexisting rhinitis, polysensitization and perennial exposure to allergens.

## Conclusions

The general conclusion of this study is that long-term exposure to allergens (e.g. mites and cat) and polysensitization are the strongest risk factors for the development of multimorbidity in allergy patients. A alone appears to be a different phenotype than A in multimorbidity, especially when coexisting with AR.

## Additional file

**Additional file 1.** Results: data analysis—supplement.

## Abbreviations

A: asthma
AD: atopic dermatitis
AR: allergic rhinitis
d1: sIgE: *Dermatophagoides pteronyssinus*
Der p: *Dermatophagoides pteronyssinus*
Der f: *Dermatoiphagoides farinae*
e1: sIgE: cat
EAACI: European Academy of Allergy and Clinical Immunology
ECAP: Epidemiology of Allergic Diseases in Poland
ECRHS: European Community Respiratory Health Survey
g6: sIgE: *Phleum pratense*
ISAAC: International Study of Asthma and Allergy in Childhood
m6: sIgE: *Alternaria alternata*
OR: odds ratio
Rh: rhinitis
HDM: house dust mites
sIgE: specific immunoglobulin E
SPT: skin prick test
