# Correction to: Dissociating polysensitization and multimorbidity in children and adults from a Polish general population cohort

**DOI:** 10.1186/s13601-019-0263-x

**Published:** 2019-04-04

**Authors:** Filip Raciborski, Jean Bousquet, Andrzej Namysłowski, Edyta Krzych-Fałta, Aneta Tomaszewska, Barbara Piekarska, Piotr Samel-Kowalik, Artur Z. Białoszewski, Artur Walkiewicz, Agnieszka Lipiec, Oksana Wojas, Krzysztof Samoliński, Anna Szylling, Wojciech Zieliński, Adam Sybilski, Aleksandra Grąbczewska, Bolesław Samoliński

**Affiliations:** 1 Department of Prevention of Environmental Hazards and Allergology, Medical University of Warsaw, Warsaw, Poland; 2 MACVIA-France and Fondation FMC VI-LR, Montpellier, France; 3 Ageing and Chronic Diseases, Epidemiological and Public Health Approaches, U1168, INSERM, VIMA, Paris, France; 4 UMR-S 1168, Université Versailles St-Quentin-en-Yvelines (UVSQ), Paris, France; 5 Euforea, Brussels, Belgium; 6 Charité, Berlin, Germany; 7 Allergy and Clinical Immunology Department, Central Hospital Medical University of Warsaw, Warsaw, Poland; 8 Public Health Department, Medical University of Warsaw, Warsaw, Poland; 9 Department of Econometrics and Statistics, Warsaw University of Life Sciences, Warsaw, Poland

## Correction to: Clin Transl Allergy (2019) 9:4 10.1186/s13601-019-0246-y

Following publication of the original article [1], the authors reported that one of the authors’ name was spelled incorrectly. In this correction, the incorrect and the correct author names are shown.

Originally, the author name was published as:

- Jean Bousqet

The correct author name is:

- Jean Bousquet
