# Supplementary material for: Dissociating polysensitization and multimorbidity in children and adults from a Polish general population cohort
Source: Clin Transl Allergy. 2019 Feb 11;9:4. doi: 10.1186/s13601-019-0246-y (PMC6369558; doi:10.1186/s13601-019-0246-y)
Supplement: Supplementary file 1 — Additional file 1. Results: data analysis—supplement. [file 13601_2019_246_MOESM1_ESM.docx]

**Additional file 1.**

**Results: data analysis - supplement**


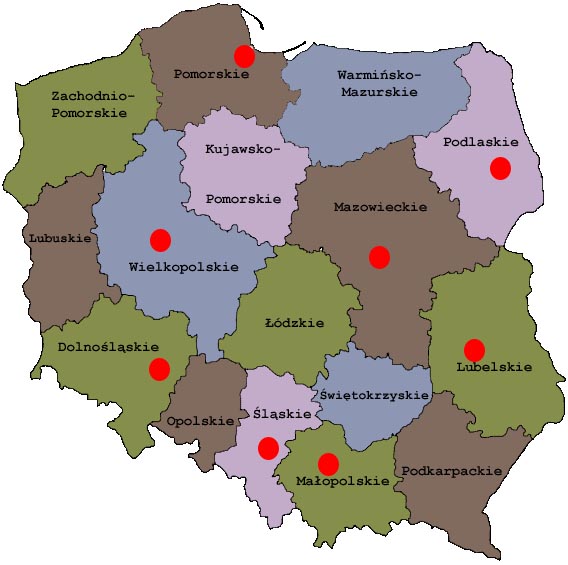


Figure 1 online. The location of the ECAP study sites on the map of Poland

Table 1 online. Characteristics of the study group – comparison between age groups

|  | 6-7 yrs | 13-14 yrs | Adults (20-44) | p-value |
| --- | --- | --- | --- | --- |
| **Allergic Diseases** |  |  |  |  |
| Asthma | 12.3% | 11.1% | 9.8% | p=0.109 |
| Asthma alone (without AR. AD) | 4.2% | 3.4% | 3.3% | p=0.438 |
| Allergic rhinitis | 25.2% | 31.2% | 30.4% | p<0.01 |
| Allergic rhinitis alone (without A. AD) | 15.2% | 20.9% | 23.0% | p<0.001 |
| Atopic dermatitis | 10.3% | 10.0% | 3.9% | p<0.001 |
| Atopic dermatitis alone (without A. AR) | 5.4% | 5.2% | 1.9% | p<0.001 |
| Food allergy | 13.7% | 11.4% | 4.9% | p<0.001 |
| Drug allergy | 3.2% | 3.8% | 5.2% | p<0.05 |
| Insect venom allergy | 1.7% | 2.1% | 3.0% | p=0.056 |
| Urticaria | 5.6% | 5.9% | 7.1% | p=0.202 |
|  |  |  |  |  |
| **Positive SPTs (≥3mm)** |  |  |  |  |
| Control | 0.6% | 0.7% | 0.9% | p=0.638 |
| Histamine | 89.3% | 94.1% | 96.0% | p<0.001 |
| Dog | 6.4% | 8.0% | 10.9% | p<0.001 |
| Cat | 9.0% | 15.4% | 14.3% | p<0.001 |
| Cladosporium | 4.2% | 4.8% | 6.6% | p<0.05 |
| Alternaria | 9.0% | 10.6% | 7.1% | p<0.01 |
| Hazel & Alder & Birch | 13.2% | 20.6% | 22.3% | p<0.001 |
| D. Pteronyssinus & D. Farinae | 18.2% | 30.3% | 29.6% | p<0.001 |
| Grass pollen & Rye grass | 17.2% | 26.7% | 24.9% | p<0.001 |
| Mugwort. Plantain | 9.7% | 20.8% | 23.2% | p<0.001 |
|  |  |  |  |  |
| **Positive sIgE (≥1 class)** |  |  |  |  |
| d1 - D pteronyssinus | 12.7% | 18.7% | 13.3% | p<0.001 |
| g6 - Phleum pratense | 8.7% | 16.7% | 13.6% | p<0.001 |
| m6 - Alternaria alternata | 4.8% | 6.1% | 2.1% | p<0.001 |
| e1 - cat | 4.3% | 8.4% | 5.7% | p<0.001 |

**Table 2 online. Prevalence of allergic diseases (confirmed diagnosis) by age group (n=3856)**

|  | 6-7 yrs | | 13-14 yrs | | 20-44 yrs | | Total | | p-value (for age categories) |
| --- | --- | --- | --- | --- | --- | --- | --- | --- | --- |
|  | n | (%) | n | (%) | n | (%) | n | (%) |  |
| A without AR, AD | 41 | (4.2%) | 37 | (3.4%) | 60 | (3.3%) | 138 | (3.6%) | p=0.438 |
| AR without A, AD | 147 | (15.2%) | 227 | (20.9%) | 416 | (23.0%) | 790 | (20.5%) | p<0.001 |
| AD without A, AR | 52 | (5.4%) | 56 | (5.2%) | 34 | (1.9%) | 142 | (3.7%) | p<0.001 |
| A and AR | 72 | (7.4%) | 76 | (7.0%) | 110 | (6.1%) | 258 | (6.7%) | p=0.352 |
| A and AD | 23 | (2.4%) | 17 | (1.6%) | 14 | (0.8%) | 54 | (1.4%) | p<0.01 |
| AD and AR | 42 | (4.3%) | 45 | (4.2%) | 30 | (1.7%) | 117 | (3.0%) | p<0.001 |
| A and AR and AD | 17 | (1.8%) | 10 | (0.9%) | 8 | (0.4%) | 35 | (0.9%) | p<0.01 |

AR - Allergic rhinitis, AD - Atopic dermatitis

*** - 15 allergens**

**** - 4 allergens**

**AR - Allergic rhinitis, AD - Atopic dermatitis**

**Figure 2 online. Prevalence of allergic multi-morbidity by age, gender, allergic diseases, and SPTs and sIgE results.**

**Table 3 online. Prevalence of positive SPTs in asthma, allergic rhinitis, atopic dermatitis alone and multimorbidity (3 and 6 mm SPT thresholds)**

| **SPT threshold** | **Diagnosis** | **No sensitisation** | **Monosensitisation** | **Polysensitisation** | **Total** |
| --- | --- | --- | --- | --- | --- |
| ≥3mm | Asthma without AR, AD | 60.9% | 13.0% | 26.1% | 100% |
|  | AR without asthma, AD | 15.9% | 21.8% | 62.3% | 100% |
|  | AD without asthma, AR | 53.5% | 23.9% | 22.5% | 100% |
|  | Allergic multimorbidity | 13.6% | 15.3% | 71.0% | 100% |
| ≥6mm | Asthma without AR, AD | 76.8% | 12.3% | 10.9% | 100% |
|  | AR without asthma, AD | 45.2% | 28.9% | 25.9% | 100% |
|  | AD without asthma, AR | 84.5% | 11.3% | 4.2% | 100% |
|  | Allergic multimorbidity | 41.2% | 25.1% | 33.7% | 100% |

**AR - Allergic rhinitis, AD - Atopic dermatitis**

**Table 4 online. Association between allergen sensitization for** ≥**3 mm and** ≥**6 mm SPT threshold and allergic diseases**

|  |  | |  | |  | | **95% CI** | |  | |  | |
| --- | --- | --- | --- | --- | --- | --- | --- | --- | --- | --- | --- | --- |
| **Diagnosis** | **SPT threshold** | **No of positive SPTs** | | **OR** | | **Lower** | | **Upper** | | **N*** | | **Total N** |
| A alone | ≥3mm | 1 | | 0.787 | | 0.469 | | 1.320 | | 102 | | 2741 |
|  |  | 2 | | 0.703 | | 0.350 | | 1.413 | | 93 | | 2483 |
|  |  | 3 | | 0.942 | | 0.467 | | 1.897 | | 93 | | 2403 |
|  |  | 4 | | 0.796 | | 0.343 | | 1.848 | | 89 | | 2350 |
|  |  | 5 | | 0.829 | | 0.331 | | 2.074 | | 89 | | 2312 |
|  |  | ≥6 | | 0.900 | | 0.411 | | 1.974 | | 91 | | 2357 |
| AR alone | ≥3mm | 1 | | 6.749 | | 5.240 | | 8.693 | | 298 | | 2741 |
|  |  | 2 | | 8.713 | | 6.518 | | 11.648 | | 240 | | 2483 |
|  |  | 3 | | 11.698 | | 8.571 | | 15.965 | | 229 | | 2403 |
|  |  | 4 | | 16.127 | | 11.533 | | 22.551 | | 222 | | 2350 |
|  |  | 5 | | 14.160 | | 9.839 | | 20.380 | | 198 | | 2312 |
|  |  | ≥6 | | 18.756 | | 13.461 | | 26.134 | | 233 | | 2357 |
| AD alone | ≥3mm | 1 | | 1.697 | | 1.120 | | 2.570 | | 110 | | 2741 |
|  |  | 2 | | 1.050 | | 0.565 | | 1.953 | | 88 | | 2483 |
|  |  | 3 | | 0.806 | | 0.367 | | 1.768 | | 83 | | 2403 |
|  |  | 4 | | 0.435 | | 0.136 | | 1.392 | | 79 | | 2350 |
|  |  | 5 | | 1.111 | | 0.476 | | 2.592 | | 82 | | 2312 |
|  |  | ≥6 | | 0.562 | | 0.203 | | 1.552 | | 80 | | 2357 |
| Allergic multimorbidity | ≥3mm | 1 | | 4.483 | | 3.015 | | 6.666 | | 104 | | 2741 |
|  |  | 2 | | 8.768 | | 5.846 | | 13.150 | | 104 | | 2483 |
|  |  | 3 | | 11.881 | | 7.841 | | 18.003 | | 102 | | 2403 |
|  |  | 4 | | 12.051 | | 7.729 | | 18.791 | | 91 | | 2350 |
|  |  | 5 | | 18.906 | | 12.118 | | 29.497 | | 96 | | 2312 |
|  |  | ≥6 | | 17.705 | | 11.671 | | 26.859 | | 107 | | 2357 |
| A alone | ≥6mm | 1 | | 0.936 | | 0.556 | | 1.576 | | 123 | | 3466 |
|  |  | 2 | | 1.709 | | 0.924 | | 3.161 | | 118 | | 3161 |
|  |  | 3 | | 0.696 | | 0.218 | | 2.227 | | 109 | | 3079 |
|  |  | ≥4 | | – | | – | | – | | 106 | | 3030 |
| AR alone | ≥6mm | 1 | | 5.980 | | 4.861 | | 7.356 | | 585 | | 3466 |
|  |  | 2 | | 8.136 | | 6.038 | | 10.962 | | 463 | | 3161 |
|  |  | 3 | | 8.203 | | 5.629 | | 11.954 | | 420 | | 3079 |
|  |  | ≥4 | | 7.720 | | 4.770 | | 12.496 | | 393 | | 3030 |
| AD alone | ≥6mm | 1 | | 0.773 | | 0.455 | | 1.313 | | 136 | | 3466 |
|  |  | 2 | | 0.238 | | 0.058 | | 0.969 | | 122 | | 3161 |
|  |  | 3 | | 0.823 | | 0.299 | | 2.268 | | 124 | | 3079 |
|  |  | ≥4 | | – | | – | | – | | 120 | | 3030 |
| Allergic multimorbidity | ≥6mm | 1 | | 4.111 | | 3.102 | | 5.447 | | 238 | | 3466 |
|  |  | 2 | | 7.338 | | 5.172 | | 10.412 | | 204 | | 3161 |
|  |  | 3 | | 7.600 | | 4.940 | | 11.691 | | 182 | | 3079 |
|  |  | ≥4 | | 15.103 | | 9.163 | | 24.893 | | 179 | | 3030 |

*Number of subjects with confirmed diagnosis.
